# Evaluation of the accuracy of ChatGPT in answering asthma-related questions

**DOI:** 10.36416/1806-3756/e20240388

**Published:** 2025-07-22

**Authors:** Bruno Pellozo Cerqueira, Vinicius Cappellette da Silva Leite, Carla Gonzaga França, Fernando Sergio Leitão, Sonia Maria Faresin, Ricardo Gassmann Figueiredo, Andrea Antunes Cetlin, Lilian Serrasqueiro Ballini Caetano, José Baddini-Martinez

**Affiliations:** 1. Escola Paulista de Medicina, Universidade Federal de São Paulo, São Paulo (SP) Brasil.; 2. Divisão de Pneumologia, Escola Paulista de Medicina, Universidade Federal de São Paulo, São Paulo (SP) Brasil.; 3. Divisão de Pneumologia, Universidade Estadual de Feira de Santana, Feira de Santana (BA) Brasil.; 4. Divisão de Pneumologia, Faculdade de Medicina de Ribeirão Preto, Universidade de São Paulo, Ribeirão Preto (SP) Brasil.

**Keywords:** Asthma, Artificial intelligence, Pulmonologists

## Abstract

**Objective:**

To evaluate the quality of ChatGPT answers to asthma-related questions, as assessed from the perspectives of asthma specialists and laypersons.

**Methods:**

Seven asthma-related questions were asked to ChatGPT (version 4) between May 3, 2024 and May 4, 2024. The questions were standardized with no memory of previous conversations to avoid bias. Six pulmonologists with extensive expertise in asthma acted as judges, independently assessing the quality and reproducibility of the answers from the perspectives of asthma specialists and laypersons. A Likert scale ranging from 1 to 4 was used, and the content validity coefficient was calculated to assess the level of agreement among the judges.

**Results:**

The evaluations showed variability in the quality of the answers provided by ChatGPT. From the perspective of asthma specialists, the scores ranged from 2 to 3, with greater divergence in questions 2, 3, and 5. From the perspective of laypersons, the content validity coefficient exceeded 0.80 for four of the seven questions, with most answers being correct despite a lack of significant depth.

**Conclusions:**

Although ChatGPT performed well in providing answers to laypersons, the answers that it provided to specialists were less accurate and superficial. Although AI has the potential to provide useful information to the public, it should not replace medical guidance. Critical analysis of AI-generated information remains essential for health care professionals and laypersons alike, especially for complex conditions such as asthma.

## INTRODUCTION

Artificial intelligence (AI) is a broad term referring to the ability of a computer system to simulate human intelligent behavior with a minimum of human intervention.^1^ Although the use of the term AI is currently on the rise, the term has been used since the middle of the last century.^2^

ChatGPT, a generative pre-trained transformer developed by OpenAI, is currently one of the most widely used AI tools. ChatGPT is a natural language processing model trained on a variety of text data, being capable of generating human-like responses within seconds.^3^ Its accessibility and ease of use have made it a subject of study in various fields of medicine.^4^

Asthma is one of the most common noncommunicable diseases, affecting over 300 million people worldwide. Because asthma is such a common disease, it is not unusual to hear a patient say that they have asthma on the basis of what they read on the internet, which is often superficial and inaccurate.^5^ Despite its widespread occurrence, asthma is a disease whose management is complex and involves critical steps, beginning with proper diagnosis and disease staging.^6^ This complexity often raises questions even among health care professionals, who frequently rely on internet sources for quick access to relevant information. 

Given the timeliness and relevance of this topic, the objective of the present study was to formulate questions addressing various aspects of asthma and pose them to ChatGPT, assessing the quality of the responses from two perspectives: those intended for laypersons and those intended for asthma specialists. 

## METHODS

Two of the authors of the present study developed twenty-one questions addressing various aspects of asthma and then selected seven that they considered to be the most important and most commonly asked when consulting ChatGPT (Table 1). The two aforementioned authors have extensive experience in asthma management. They formulated the questions using the GINA as a reference. To obtain the most accurate answers, the paid version of ChatGPT (version 4) was used. The questions were asked between May 3, 2024 and May 4, 2024. To ensure the uniformity of the answers provided by ChatGPT, the questions were asked in the same format, with a request for answers to be approximately two pages long, thus guaranteeing consistent content. To reflect how the general population uses large language models, specific prompts were avoided. Since ChatGPT can retain information from previous interactions, the option not to save any of the conversations was selected, and the history was deleted after each response in order to minimize potential bias. Each question was asked twice at consecutive times after the previous chat had been cleared in order to assess the reproducibility of the answers. 

**Table 1:** Asthma-related questions asked to ChatGPT.

Number	Question
1	What is asthma?
2	How to diagnose asthma?
3	How is asthma severity classified?
4	How is asthma control classified?
5	What is the pharmacological treatment for asthma?
6	Is there a cure for asthma?
7	What are the risk factors for asthmatic patients experiencing poor outcomes in the future?

After data collection, the answers were sent to specialists in pulmonology and asthma, along with a form, for independently assessing the reproducibility and quality of the answers. The experts were instructed to assess the answers on the basis of current guidelines and updates, particularly GINA guidelines. 

A total of six experts evaluated the seven answers provided by ChatGPT from two perspectives: ChatGPT answers intended for a layperson; and ChatGPT answers intended for an asthma specialist. Each answer (item) was scored from 1 to 4 on a Likert scale, as follows: 1-totally correct; 2-correct but insufficient; 3-contains information that is correct and information that is incorrect; and 4-totally incorrect. The level of agreement among the six experts was analyzed by calculating the content validity coefficient (CVC)^7^ for each item, as follows:

$$CVC=\frac{\left[\left(\frac{\Sigma x}{J}\right)\right]}{Vmx}-p$$

where *x* represents the mean scores; *J* represents the total number of judges (or experts, i.e., 6); *Vmx* represents the highest possible score; and *p* represents the bias, which was calculated as follows:

$$p={\left(\frac{1}{J}\right)}^{J}$$

**Figure 1:**
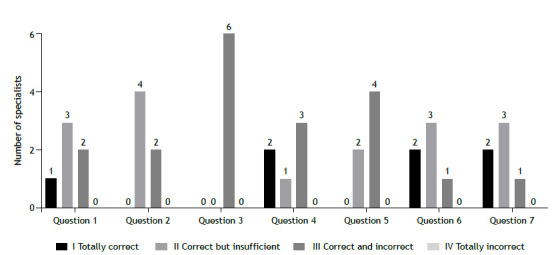
Expert opinion on the quality of ChatGPT answers from the perspective of asthma specialists.

**Figure 2:**
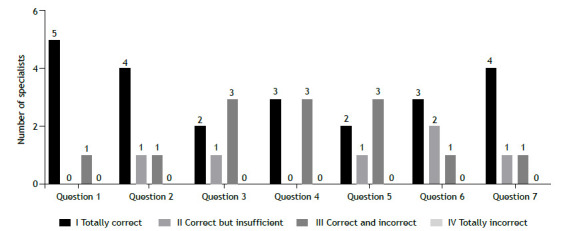
Expert opinion on the quality of ChatGPT answers from the perspective of laypersons.

As a rule, the cutoff point for acceptable item validity is 0.80 in content validity studies of scales measuring psychological phenomena. However, this is not applicable to all contexts, and we did not want to impose a fixed cutoff point in the present context. The program GraphPad Prism, version 8.0 (GraphPad Software, Inc., San Diego, CA, USA) was used in order to create Figures 1 and 2. 

## RESULTS

Regarding the reproducibility of the answers provided by ChatGPT when questions were asked repeatedly, the expert panel in the present study considered that more than 85% of the answers were consistent. Those who considered that some of the questions lacked reproducibility attributed it to variability or misclassification of certain topics. 

The results of the assessments are shown in Figure 1 (from the perspective of asthma specialists) and Figure 2 (from the perspective of laypersons). None of the experts assigned a score of 4 to any of the answers that they analyzed. From the perspective of asthma specialists, the most prevalent scores were 2 (correct but insufficient) and 3 (contains information that is correct and information that is incorrect) across all questions, with question 3 receiving the highest proportion of low scores (100% scored 3). From the perspective of laypersons, a score of 1 (totally correct) was the most common, accounting for 55% of all possible answers. Question 1 had the highest number of responses that received a score of 1 (five of six). 

Table 2 shows the level of agreement among the six experts regarding the suitability of the answers provided by ChatGPT from the perspectives of asthma specialists and laypersons. From the perspective of asthma specialists, CVC values were satisfactory for questions 6 and 7, and reasonable for questions 1 and 4. For questions 2, 3, and 5, however, the experts disagreed with regard to the suitability of the answers provided by ChatGPT. For answers analyzed from the perspective of laypersons, there was greater agreement among the experts in comparison with those assessed from the perspective of asthma specialists, with CVC values exceeding 0.8 for four items: 1, 2, 6, and 7. Lower CVC values were observed for items 3, 4, and 5. 

**Table 2:** Agreement among judges regarding the suitability of ChatGPT answers from the perspectives of asthma specialists and laypersons.

Question	CVC	
	Specialist	Layperson
1	0.71	0.92
2	0.67	0.88
3	0.50	0.71
4	0.71	0.75
5	0.58	0.71
6	0.79	0.83
7	0.79	0.88

CVC: content validity coefficient.

## DISCUSSION

The widespread availability of information on the internet has resulted in a growing reliance on knowledge sources without adequate critical analysis, even among health care professionals. This trend presents significant challenges, particularly when clinical management is complex, as is the case with asthma. 

Despite being a common condition, asthma requires a multidimensional approach for effective management. The latest GINA report, published in 2024, outlines two potential therapeutic pathways organized into steps, emphasizing the complexity of treatment.^8^ Despite the fact that most pulmonologists are well-equipped to manage disease effectively, many patients are treated by primary care physicians. This is due to the high prevalence of asthma. Primary care physicians often lack specialized and comprehensive training in asthma care, and this can impact the quality of care provided to patients. 

Reddel et al.^9^ evaluated the degree of heterogeneity in the diagnosis and management of asthma and COPD in a cohort of more than 11,000 patients. The findings revealed significant variability in diagnosis and treatment, with evidence of under or overtreatment in relation to disease severity. Notably, approximately 50% of those patients were managed in primary care, a proportion that is likely similar to or even lower than that observed in Brazil. Although there is a lack of specific data on this topic, it is plausible that primary care physicians are increasingly relying on AI tools to manage complex conditions such as asthma. 

Our analysis revealed that although ChatGPT frequently provided correct information, it also made errors or offered insufficient responses for the required level of expertise. Although ChatGPT did not achieve a CVC with consistently positive concordance values, it can still be useful if the information that it provides is critically evaluated. 

The present study also focused on evaluating content generated by ChatGPT from the perspective of a layperson, given the growing trend of self-diagnosis and the increasing search for health information online. Before the widespread use of AI, Google was the primary source for such inquiries, being informally referred to as “Dr. Google.” Today, given the easy access to AI-powered tools, it is inevitable that patients will turn to such platforms for information. In a study involving 607 participants, approximately 80% expressed willingness to use ChatGPT for self-diagnosis.^10^ This finding underscores the importance of assessing the accuracy of the content that ChatGPT provides to laypersons. The present study shows that, from the perspective of a layperson, the responses were mostly correct but often insufficient, suggesting the potential of ChatGPT to inform the public. However, it is crucial to emphasize that ChatGPT should not be used as a substitute for a medical diagnosis. 

Several studies have evaluated the role of ChatGPT in medical specialties such as urology and oncology, yielding results similar to ours.^11^ ^,^ ^12^ Yeo et al.^11^ assessed the quality of ChatGPT responses using brief prompts, with evaluations carried out by two specialists. They found that although most responses were partially correct, they often lacked completeness and included accurate and inaccurate information. These findings are consistent with those of our study. Notably, the accuracy of the responses varied by topic, such as general concepts, diagnosis, and treatment, with higher accuracy observed in specific areas. This was also evident in our analysis. Ayers et al.^13^ investigated whether ChatGPT could provide responses comparable in quality and empathy to those of physicians. Interestingly, 80% of patients preferred the responses generated by ChatGPT, suggesting good accuracy and a patient-friendly approach to addressing their questions. 

In a recent study conducted in Denmark, the authors analyzed 26 asthma-related questions.^14^ The results showed that ChatGPT could provide adequate responses, albeit with some inconsistencies. However, because the aforementioned study was a brief report, it lacked methodological details, such as the version of the software used and the dates when the queries were made. Additionally, there was no evaluation of the responses from the perspective of the lay public. 

In our study, we observed a significant inconsistency in the responses provided by ChatGPT, particularly regarding the classification of asthma severity (question 3). According to the American Thoracic Society/European Respiratory Society task force^15^ and several international guidelines, asthma severity should currently be classified retrospectively, on basis of the level of treatment required to achieve disease control and prevent exacerbations. In other words, severity is determined by the dose of inhaled corticosteroids needed to manage symptoms rather than by the mere presence or intensity of those symptoms. However, ChatGPT incorrectly described the classification as follows: “The classification of asthma severity is generally divided into four categories: mild intermittent, mild persistent, moderate persistent, and severe persistent. This categorization helps determine the appropriate treatment regimen and is based on the frequency and intensity of symptoms, nighttime awakenings, the use of short-acting beta-agonists for quick relief, and the impact on normal activities.” For example, GINA defines severe asthma as asthma that remains uncontrolled despite high doses of inhaled corticosteroids combined with long-acting bronchodilators, or that requires chronic use of oral corticosteroids. In contrast, mild asthma is characterized by symptom control achieved with low doses of inhaled corticosteroids, such as budesonide at a maximum daily dose of 400 µg. Those definitions are different from those provided by ChatGPT. 

There was a high level of consistency in the evaluations made by specialists regarding certain responses. For example, question 6 (“Is there a cure for asthma?”) received widespread agreement among reviewers. The response provided by ChatGPT was as follows: “There is currently no cure for asthma. However, the condition can be effectively managed through a combination of treatments and strategies, allowing many individuals with asthma to lead normal and active lives.” This is consistent with current medical understanding and was deemed accurate and appropriate by most of the asthma specialists in the present study. 

The present study has some limitations. One major limitation is the use of short prompts. Although more specific prompts generally produce higher-quality responses, we chose to use concise commands to replicate everyday usage scenarios. However, this decision may have compromised the quality of the generated content to some extent. Additionally, the evaluation of the content was subjective, given that it involved different specialists conducting the assessments. To reduce this bias, we calculated the CVC and included a substantial number of reviewers, thus strengthening our analysis. Another significant limitation is related to the evaluation of layperson-oriented content by specialists. To assess public understanding more accurately, a different methodology would be necessary, although it would not align with the current study design. The objective of our evaluation was to have experts review the content based on what they consider essential for patients to understand about the disease. Finally, since ChatGPT is a constantly evolving model, the responses provided at a later time may vary. 

In conclusion, ChatGPT has the potential to generate informative responses for general audiences, with satisfactory agreement among reviewers in certain areas. However, when evaluated by specialists-particularly regarding more complex clinical concepts-the responses were often interpreted with greater variability and deemed less accurate. Given the known risk of AI-generated “hallucinations,” which are plausible but incorrect or misleading pieces of information, it is crucial to emphasize that language models such as ChatGPT should not be used as the sole source of health information. This caution is especially important for managing diseases such as asthma, which require individualized and nuanced care. To promote the safe and effective use of AI tools in clinical practice and health education, we recommend the following: use AI-generated information as a complementary tool rather than a replacement for professional medical advice; health care professionals should guide and supervise the use of these tools, especially when patients or caregivers are involved; developing frameworks for validating and curating AI-generated content may help ensure alignment with current clinical guidelines and reduce the spread of misinformation; and educational strategies should include digital health literacy to empower users to critically evaluate the reliability of AI responses. 

Ultimately, although ChatGPT can serve as a starting point for health-related inquiries, human oversight remains essential to maintain the quality and safety of medical information. 
